# Effects of different dietary methionine and cysteine ratios on growth performance and intestinal development of broilers from brain-gut peptide secretion perspective

**DOI:** 10.5713/ab.250787

**Published:** 2026-02-06

**Authors:** Zhihui Chen, Yang Zhao, Haoliang Chai, Dexin Zhao, Liangmei Xu, Teng Teng

**Affiliations:** 1 College of Animal Science and Technology, Northeast Agricultural University, Harbin, China

**Keywords:** Brain Gut Peptide, Broiler Hypothalamus, Ileum, Methionine-to-cysteine Ratio, Proteomics

## Abstract

**Objective:**

The objective of this study is to investigate the nutritional metabolism and growth development of broiler chickens under different methionine (Met)-to-cysteine (Cys) ratios, with a focus on proteomic alterations in the hypothalamus and ileum to elucidate potential regulatory mechanisms.

**Methods:**

A total of 216 one-day-old Arbor Acres broiler chicks were randomized into three groups: low (LMCR, 0.34%:0.56%), medium (MMCR, 0.45%:0.45%), and high (HMCR, 0.53%:0.37%) Met-to-Cys ratios, with total sulfur amino acids fixed at 0.9%. Each group comprised 6 replicates of 12 chicks, and the experiment spanned 21 days. On day 22, the birds were fasted for 12 hours before slaughter to collect serum, hypothalamus and ileum samples for comprehensive analysis of metabolic hormones, neurotransmitters and proteomic profiles.

**Results:**

Results demonstrated that HMCR improved average daily gain (p<0.05), reduced feed conversion ratio (p<0.05), and enhanced protein/lipid utilization efficiency. Mechanistically, HMCR promoted hypothalamic neurodevelopment, downregulated key components of the protein kinase A signaling pathway (p<0.05) and leptin expression (p<0.001), while upregulating growth hormone and gastrointestinal hormone secretion. Through the hypothalamic-hormonal-ileal axis, HMCR modulated ileal cell physiology by upregulating inositol 1,4,5-trisphosphate receptors (p<0.05) and phosphoinositide 3-kinase (p<0.05), thereby enhancing ileal villus morphology and nutrient absorption capacity. The improved ileal structure further augmented energy utilization efficiency (p<0.05).

**Conclusion:**

In conclusion, a dietary Met:Cys of 0.53%:0.37% optimizes broiler growth performance by coordinately regulating metabolic pathways in the hypothalamus and ileum, as well as modulating brain-gut peptide signaling. These findings provide a scientific foundation for formulating sulfur amino acid-optimized diets to enhance poultry productivity and feed efficiency.

## INTRODUCTION

Methionine (Met) serves as the primary limiting amino acid in corn-soybean meal-based broiler diets, playing an irreplaceable role in protein synthesis, sulfur metabolism, and DNA methylation owing to its unique sulfur-containing thioether side chain and labile methyl group [1]. Beyond metabolic functions, Met protects vital organs such as the brain, liver, and muscles against oxidative damage, with dietary deficiency shown to impair broiler growth performance, antioxidant capacity, and ileal mucosal immunity [2]. Cysteine (Cys), essential for keratin synthesis to maintain feather and skin integrity, is endogenously derived from Met. However, the optimal dietary methionine-to-cysteine ratio (MCR) in poultry nutrition remains undefined, presenting a critical knowledge gap in feed formulations. Current nutritional guidelines offer conflicting recommendations: the National Research Council (NRC) suggests that Cys should constitute 43% to 47% of total sulfur amino acids, whereas Wheeler and Latshaw proposed a superior effect at 38% to 43% Cys in total sulfur amino acids [3]. Exceeding 50% Cys in total sulfur amino acids triggers preferential utilization over Met, disrupting growth and development. This discrepancy underscores the urgency to define the MCR for optimizing broiler nutritional efficiency.

The hypothalamus acts as a central regulator of appetite and energy homeostasis, secreting hormones that modulate gastrointestinal function via the brain-gut axis. Key gut-brain peptides such as vasoactive intestinal peptide (VIP), ghrelin (GHRL), neuropeptide Y (NPY), and leptin (LEP) interact with G-protein-coupled receptors (GPCR) to coordinate nutrient absorption and metabolism. Over 40 GPCR have been implicated in pathways regulating food intake and energy balance, highlighting the complex signaling networks involved [4]. The aim of this study was to characterize the impact of different MCR on the growth performance and nutritional metabolism of broilers, with a particular focus on proteomic changes in the hypothalamus and ileum. By studying hormonal signaling and cellular mechanisms, we sought to identify the optimal MCR for improved growth efficiency and to inform precise poultry nutrition.

## MATERIALS AND METHODS

### Animals and experimental design

For this experiment, 216 healthy, one-day-old Arbor Acres (AA) broiler chicks were randomly divided into three groups of six replicates of 12 chickens each. Each group of broiler chickens consisted of equal numbers of males and females. The initial weight of broiler chickens was 43.0±0.5 g. The experimental period lasted 21 days. Basal rations were formulated based on metabolizable energy, crude protein (CP) and other general nutrient levels according to the broiler feeding standards of the Chinese Ministry of Agriculture and the NRC [5,6]. Various nutrients of the feed were determined (Table 1). The feed formulation for the pre-feeding period is presented in Supplement 1. Met (L-methionine, 99% purity; Sigma-Aldrich) and Cys (L-cysteine, 99% purity; Sigma-Aldrich) were supplemented to the basal diet. The total sulfur-containing amino acids in the diets of both the experimental groups was 0.9%. The ratios of Met to Cys on an air-dry matter basis in the diet were 0.34%:0.56% (low Met-to-Cys ratio, LMCR), 0.45%:0.45% (middle Met-to-Cys ratio, MMCR), and 0.53%:0.37% (high Met-to-Cys ratio, HMCR). Nutrient levels were kept consistent across the diet for all groups, except for the varying levels of Met and Cys.

### Experimental management

The experiment was conducted at the Acheng Campus Experimental Base of Northeast Agricultural University. The broilers were housed in three-tier battery cages made of galvanized steel wire. Each cage replicate measured 100 cm in length, 80 cm in width, and 45 cm in height, providing a floor area of 0.80 m^2^ per replicate (stocking density: 15 birds/m^2^). Each cage was equipped with two nipple drinkers and a long trough feeder extending the full length of the cage front (100 cm) to ensure unrestricted access to water and feed. Prior to initiation, the poultry house was thoroughly cleaned, fumigated, and disinfected in a sealed environment, followed by adequate ventilation. For the first week, the temperature was maintained at 31°C to 32°C. Thereafter, the temperature was reduced by 2°C each week for seven days, ensuring it did not fall below 23°C. From one week of age, the humidity was set to between 65% and 70%. From the second week onwards, the humidity level was maintained at 50% to 60%. The broiler chicks had ad libitum access to water throughout the experiment. The main experiment was preceded by a one-week pre-feeding period. Experimental feed was gradually introduced, starting with a 25% daily increase from days 5 to 8, with full feed transition achieved by day 8. During the experimental period, the broiler chickens were given an ad libitum access to feed, which was recorded daily. Lighting conditions combined natural and artificial sources to optimize growth and welfare for the broilers.

### Sample collection

On day 22 of the experimental period, the broiler chicken with the body weight closest to the average was selected from each replicate group, fasted for 12 hours, and then slaughtered. Each group consisted of six duplicates, with an equal proportion of females and males. The hypothalamus and ileum of the chickens were collected. Blood samples were collected from the neck into anticoagulant-free tubes. Serum was obtained by centrifuging the blood at 3,000×g for 10 minutes at 4°C. The samples were then stored at −80°C for subsequent analysis. In addition, approximately 0.2 cm of ileum and 1 to 2.5 mm^3^ of hypothalamic tissue was obtained from each chicken. Immediately after slaughter, the skull was opened, and the hypothalamus was dissected according to the stereotaxic atlas of the chicken brain [7,8]. Briefly, transverse incisions were made at the apex of the optic chiasm and the anterior margin of the mammillary bodies. Bilateral incisions (2 mm) were then made along the midline to extract the entire hypothalamus. Concurrently, the abdominal cavity was opened. A segment measuring 2 centimeters of the ileum (defined as the section extending from Meckel’s diverticulum to the ileo-cecal junction) was excised. The intestinal segment was then opened longitudinally and flushed with ice-cold, sterile phosphate-buffered saline (PBS) to remove digesta. These samples were placed in 4% paraformaldehyde solution for fixation.

### Determination of the nutrients in feed

The feeds were analyzed for organic matter (OM) by GB/T 6438-2022 [9], CP GB/T 6432-2018 [10], calcium GB/T 6436-2018 [11] and phosphorus GB/T 6437-2018 [12] levels were determined according to the Chinese national standards. Metabolizable energy was a calculated value.

### Growth performance

The body weight of each group of broiler chickens was recorded at the beginning of the experiment, and then again on day 21. Feed intake was recorded daily and the amount of remaining feed was calculated weekly. Average daily gain (ADG) is calculated by dividing the weight gain during the experimental period by the number of days in the experiment. The feed conversion ratio (FCR) was calculated by dividing the average feed intake by the ADG. Feed intake was recorded daily and remaining feed was calculated weekly. For evaluating growth performance, the entire population of each group of broilers was included in the statistical analysis (six replicates of 12 broilers per group).

### Measurement of blood biochemistry, gastrointestinal hormone and neurotransmitter indicators

The concentrations of serum total protein (TP), albuminous protein (ALB), urea nitrogen (BUN), cholesterol (CHO), glucose (GLU), total triglyceride (TG), high-density lipoprotein (HDL), and low-density lipoprotein (LDL) were analyzed using an automated biochemistry analyzer (Beckman Coulter). Serum and hypothalamic tissue concentrations of growth hormone releasing peptide (GHRL; H333; Jiancheng Biotechnology), LEP (H174; Jiancheng Biotechnology), VIP (H167; Jiancheng Biotechnology), gastric inhibitory peptide (GIP. H513; Jiancheng Biotechnology), NPY (H167; Jiancheng Biotechnology), and 5-hydroxytryptophan (5-HT; H104; Jiancheng Biotechnology) were measured using ELISA kits produced by Nanjing Jiancheng Biotechnology. All assays were performed according to the manufacturer’s instructions and absorbance values were measured using a multi-mode microplate reader (Deken GENIOS). Six replicates of one broiler chick per replicate were selected for each group for the experiment.

### Histopathology and ultrastructure analysis

The ileum and hypothalamus samples were fixed, then embed them in paraffin and section them. The sections were then stained with haematoxylin and eosin (H&E) and visualized using the neural cell development marker bromodeoxyuridine (BrdU). The sections were then examined under a Nikon Eclipse Ci-L microscope (Nikon Corporation) at 200× magnification.

### Immunofluorescence staining of frozen hypothalamic tissue sections

After fixation, the hypothalamic tissue was rinsed with PBS and dehydrated overnight. Following the embedding process, the tissue sections were visualized using a light microscope. The BrdU antibody (Beijing Bioscience Biotech) was diluted and added to PBST that has 10% BSA and 0.1% Triton in it. The samples were washed three times with PBST (0.1% Triton) for 10 minutes each time. Sections were incubated at room temperature in the dark with DAPI (1:500 dilution) and goat anti-rabbit IgG-HRP (1:3,000 dilution) for 2–3 hours (the dilution buffer contained 10% BSA). After removing extra liquid, the samples were mounted with special glue and stored at 4°C. Finally, they were observed using a fluorescence microscope that can see glowing particles.

### Quantitative reverse transcription polymerase chain reaction analysis

Total RNA was extracted from the hypothalamus using TRIzol Reagent, which was produced by Takara Biomedical Technology. Total RNA was then reverse transcribed into cDNA using a PrimeScript RT reverse transcription kit RR092A (Takara Biomedical Technology). The resulting cDNA was then subjected to reverse transcription quantitative polymerase chain reaction (RT-qPCR) analysis using SYBR Premix Ex Taq RR420A (Takara Biomedical Technology) to determine mRNA expression levels. The relative mRNA expression levels of target genes were normalized using GAPDH as an internal reference and defined using the 2^−ΔΔCt^ method. All primer information is summarized in Table 2.

### Proteomics

Three biological replicate samples were selected for iTRAQ analysis in each experimental group. Following the manufacturer’s operating procedures, the labelled samples were mixed and separated into 12 components using a high-performance liquid chromatography (HPLC) system (Thermo Fisher Scientific DIONEX). A Durashell C18 (5 μm, 100 A, 4.6 mm× 250 mm) chromatographic column was used. Liquid chromatography–electrospray ionisation tandem mass spectrometry (LC–ESI–MS/MS) analysis was performed using a TripleTOF 5600 Plus system (Agilent Technologies). Proteins were considered to be differentially expressed when the iTRAQ ratio was equal to or greater than 1.2 or less than 0.83. Statistical significance was determined using a t-test, with p<0.05 considered statistically significant. Gene Ontology (GO) annotation and Kyoto Encyclopedia of Genes and Genomes (KEGG) pathway enrichment analysis were employed to explore functional subcategories and metabolic pathways associated with these proteins.

### Statistical analysis

The present study commenced with the execution of normality tests and analysis of variance (ANOVA) on the data. Subsequently, one-way ANOVA was conducted using SPSS 22.0 software, followed by Tukey’s multiple comparison test for further analysis. The visualization of all data was conducted utilizing GraphPad Prism software (ver. 8.0; GraphPad Software). The presentation of experimental data is as the mean± standard error of the mean (SEM), with * indicating p<0.05 and ** indicating p<0.01.

## RESULTS

### Effect of methionine-to-cysteine ratio on growth performance and related hormones in broiler chicks

**Figure 1:**
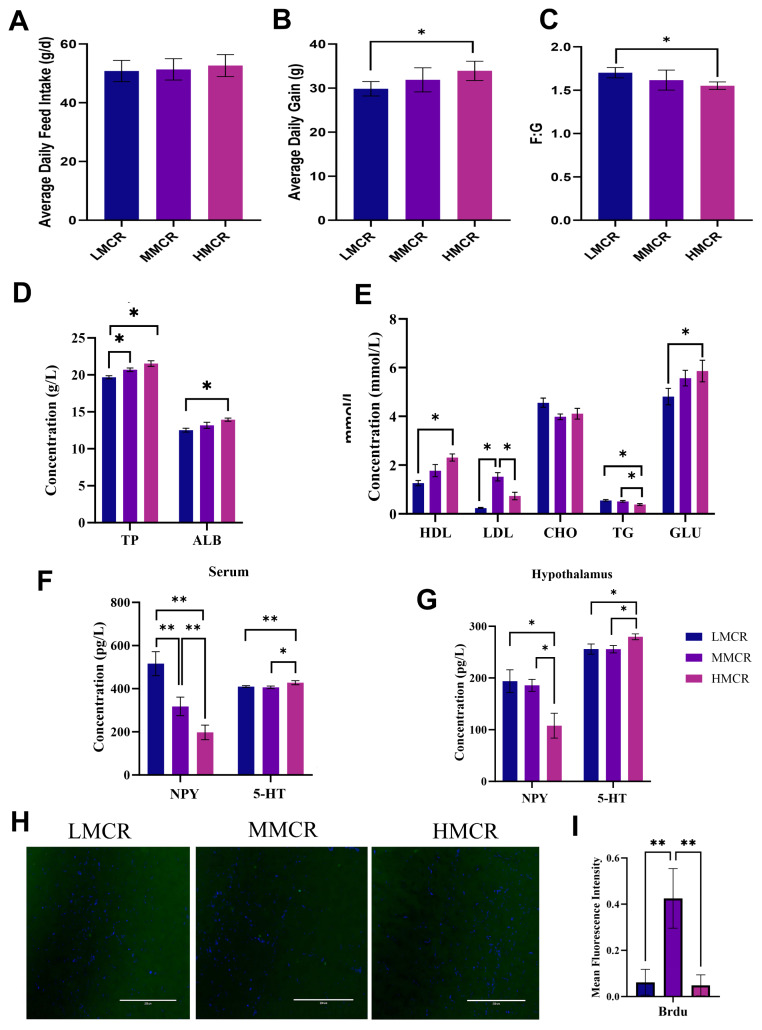
High Met-to-Cys ratio (MCR) can enhance the growth metabolism of broiler chickens and promote their development. (A) Effect of MCR on average daily food intake (ADFI) of 21-day-old broilers. (B) Effect of MCR on average daily gain (ADG) of 21-day-old broilers (n = 6; twelve bird per replicate). (C) Effect of MCR on feed conversion ratio (FCR) of 21-day-old broilers (n = 6; twelve bird per replicate). (D) Effect of sulfur amino acids ratio on albuminous protein (ALB) and total protein (TP) in serum of 21-day-old broilers (n = 6). (E) Effect of sulfur amino acids ratio on high-density lipoprotein (HDL), low-density lipoprotein (LDL), cholesterol (CHO), total triglyceride (TG), glucose (GLU) in serum of 21-day-old broilers (n = 6). (F) Effects of different MCR on neuropeptide Y (NPY) and 5-hydroxytryptamine (5-HT) in serum of 21-day-old broilers (n = 6). (G) Effects of different ratios of Met and Cys on NPY and 5-HT in hypothalamus of 21-day-old broilers (n = 6). (H, I) Representative images (H) and quantitative analysis (I) of Brdu IHC in the hypothalamus, n = 3; scale bar, 20 μm. * p<0.05; ** p<0.01. LMCR, low Met-to-Cys ratio; MMCR, middle Met-to-Cys ratio; HMCR, high Met-to-Cys ratio; BrdU, bromodeoxyuridine; Met, methionine; Cys, cysteine.

Firstly, an evaluation was conducted of the effect of different MCR in feed on the growth performance of broiler chickens. Compared to the LMCR, the ADG increased with the high MCR (HMCR) (Figure 1A, p<0.05), and the FCR decreased (Figure 1B, p<0.05). As a consequence of the modified growth performance exhibited by broiler chickens under varying MCR conditions, indicators associated with nutrient metabolism were measured in their serum (Figures 1D, 1E). The TP content was found to be higher in the HMCR than in the LMCR and MMCR (p<0.05). The serum albumin (ALB) content of the HMCR was higher than that of the LMCR (p< 0.05). On day 21, the HDL content of the HMCR serum was higher than that of the LMCR serum (Figure 1E, p<0.05). Triglyceride content was lower in the HMCR than in the LMCR and MMCR (Figure 1E, p<0.05). GLU content was higher in the HMCR than in the LMCR (Figure 1E, p<0.05).

The effects of different MCR on serum and hypothalamic neurotransmitters in 21-day-old broiler chickens were measured. Changes in dietary MCR affected the levels of NPY and serotonin (5-HT) in serum. The NPY levels in the LMCR and MMCR groups were higher than in the HMCR group (Figure 1F, p<0.01). Conversely, 5-HT levels in hypothalamic tissue were higher in the HMCR group than in the LMCR and MMCR groups (Figure 1F, p<0.05). Levels of NPY in the LMCR and MMCR groups were higher than in the HMCR group (Figure 1G, p<0.05). In contrast, an elevation in 5-HT levels was observed in the hypothalamic tissue of the HMCR group when compared to the LMCR and MMCR groups (Figure 1G, p<0.05). Figure 1H shows the effects of different MCR on the development of hypothalamic neural cells in 21-day-old broiler chickens. Fluorescent microscopy revealed that expression of the developmental marker BrdU in the hypothalamus increased with rising MCR in the diet. This finding suggests that elevated dietary MCR may promote hypothalamic development in broiler chickens.

### Different methionine-to-cysteine ratios affect hypothalamic protein composition

**Figure 2:**
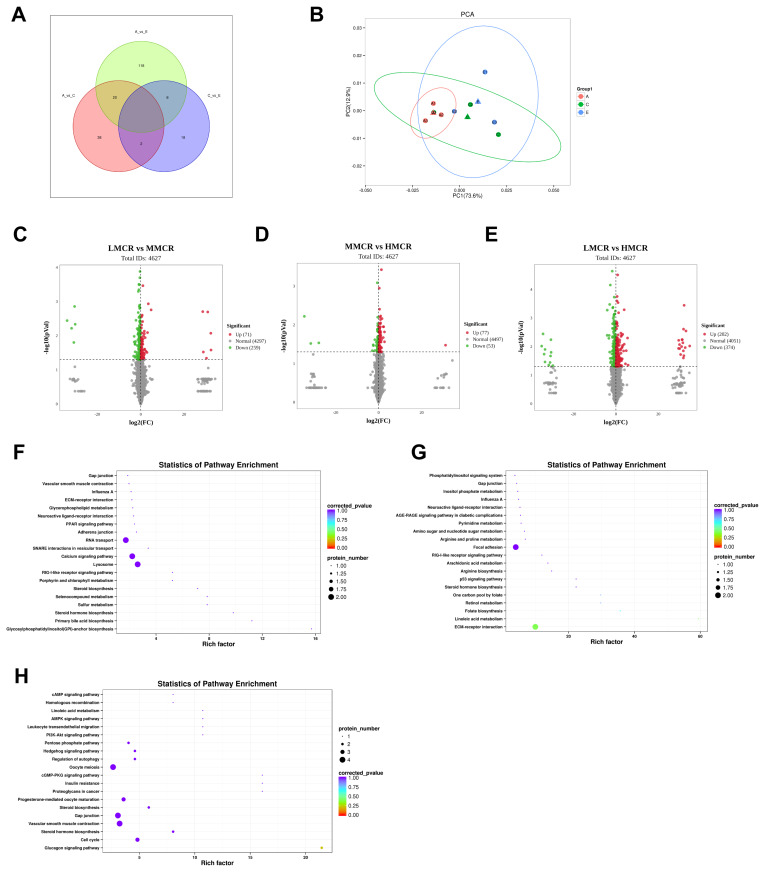
Proteomic analysis of the hypothalamus in broiler chickens under different MCR conditions. (A) Analysis of differential proteins by Venn (n = 3). (B) Analysis of differential proteins by PCA (n = 3). Red indicates upward adjustments, green indicates downward adjustments. (C–E) GO functional classification and clustering results of differential expression proteins (n = 3). (F–H) KEGG functional classification and clustering results of differential expression proteins (n = 3). * p<0.05; ** p<0.01. LMCR, low Met-to-Cys ratio; MMCR, middle Met-to-Cys ratio; HMCR, high Met-to-Cys ratio; MCR, methionine-to-cysteine ratio; GO, Gene Ontology; KEGG, Kyoto Encyclopedia of Genes and Genomes; Met, methionine; Cys, cysteine.

As illustrated in the Venn diagram (Figure 2A), a total of 230 differentially expressed proteins were identified between the LMCR, MMCR, and HMCR groups. Specifically, a total of 60 differentially expressed proteins were identified between the LMCR and MMCR, with 26 being expressed at a higher level and 34 at a lower level. There were 144 differentially expressed proteins between LMCR and HMCR, and 26 differentially expressed proteins between MMCR and HMCR. Given the regulatory effect of Met on endocrine function, we explored the protein metabolism within the hypothalamus, which served as the body’s hormonal regulation center, under different MCR, as well as the functions of differentially expressed proteins. We identified key proteins and important pathways by referencing the GO and KEGG databases. Figures 2C–2E shows the volcano plot of differentially expressed proteins.

### Functional annotation of Kyoto Encyclopedia of Genes and Genomes database of differential proteins under different methionine-to-cysteine ratio

The analysis of the differential proteins between LMCR and MMCR (Figure 2I) revealed the names of the differential proteins and their associated KEGG pathways. A total of 35 metabolic pathways were involved, including 12 pathways related to cellular processes such as apoptosis, oocyte meiosis, lysosome, and adherens junctions (Supplement 2). These findings aligned with the GO functional annotation of the relevant cellular functions. Furthermore, eight pathways were identified as being associated with metabolic processes, twelve pathways with environmental information processes, and the remaining pathways with genetic information processes. For the differential proteins between MMCR and HMCR (Figure 2J). A total of 24 KEGG pathways were involved, with 12 pathways related to metabolic processes (Supplement 3). Among these 24 KEGG pathways, three pathways were associated with cytochrome P-2 (Cyp-2). Finally, we analyzed the differential proteins between LMCR and HMCR (Figure 2K), identified a total of 50 KEGG pathways were involved, with 32 pathways associated with cellular processes (Supplement 4). Notably, 11 of the pathways involved were related to protein kinase A (PKA).

### Increasing the methionine-to-cysteine ratio inhibited leptin activity by affecting the protein kinase A pathway, thereby promoted the synthesis of growth hormone

**Figure 3:**
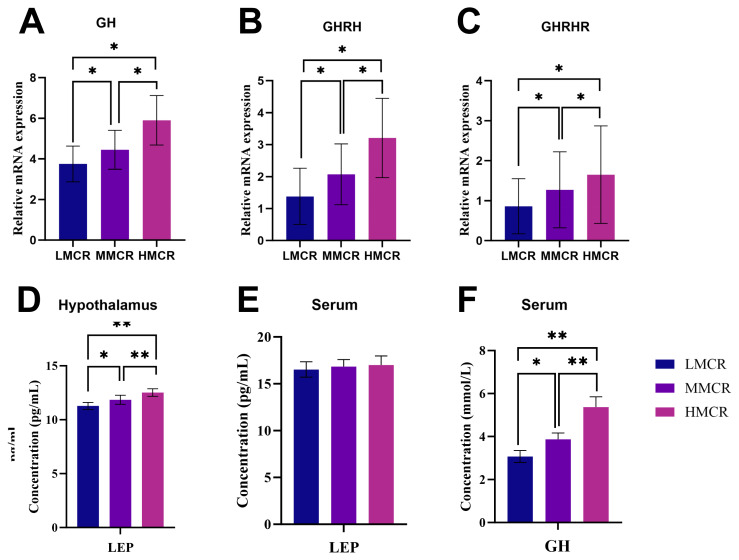
High Met-to-Cys ratio (MCR) levels inhibit leptin (LEP)’s effects, and promote the expression of growth hormone (GH) (n = 6). (A–C) Effects of different MCRs on GH, GHRH, GHRHR related gene expression in hypothalamus of 21-day-old broilers (n = 6). (D) Effects of different MCRs on LEP in hypothalamus of 21-day-old broilers (n = 6). (E) Effects of different MCRs on LEP in serum of 21-day-old broilers (n = 6). (F) Effect of MCRs on GH in serum of 21-day-old broilers (n = 6). * p<0.05; ** p<0.01. LMCR, low Met-to-Cys ratio; MMCR, middle Met-to-Cys ratio; HMCR, high Met-to-Cys ratio; Met, methionine; Cys, cysteine.

Based on the proteomics results, we found that the PKA protein played a key role in hypothalamic protein metabolism. This further confirmed the results of the proteomics analysis. The effect of different MCRs on the expression of growth hormone-related genes in the hypothalamus of broiler chickens was also measured. The concentration of growth hormone (GH) in the serum increased with the MCR concentration, with the HMCR being higher than the LMCR and MMCR groups (p<0.05), and the MMCR being higher than the LMCR group (p<0.05). The GHRH, GHRHR and GH gene expression in the hypothalamus of broiler chickens all increased with the rise in MCRs (Figures 3F–3H). In the hypothalamus of broiler chickens, GHRH, GHRHR and GH gene expression was found to be higher in the HMCR group than in the LMCR and MMCR groups (p<0.05). Furthermore, expression was found to be higher in the MMCR group than in the LMCR group (p<0.05).

### Increasing the methionine-to-cysteine ratio improves the ileum morphology in broiler chickens and promotes the secretion of gastrointestinal hormones

**Figure 4:**
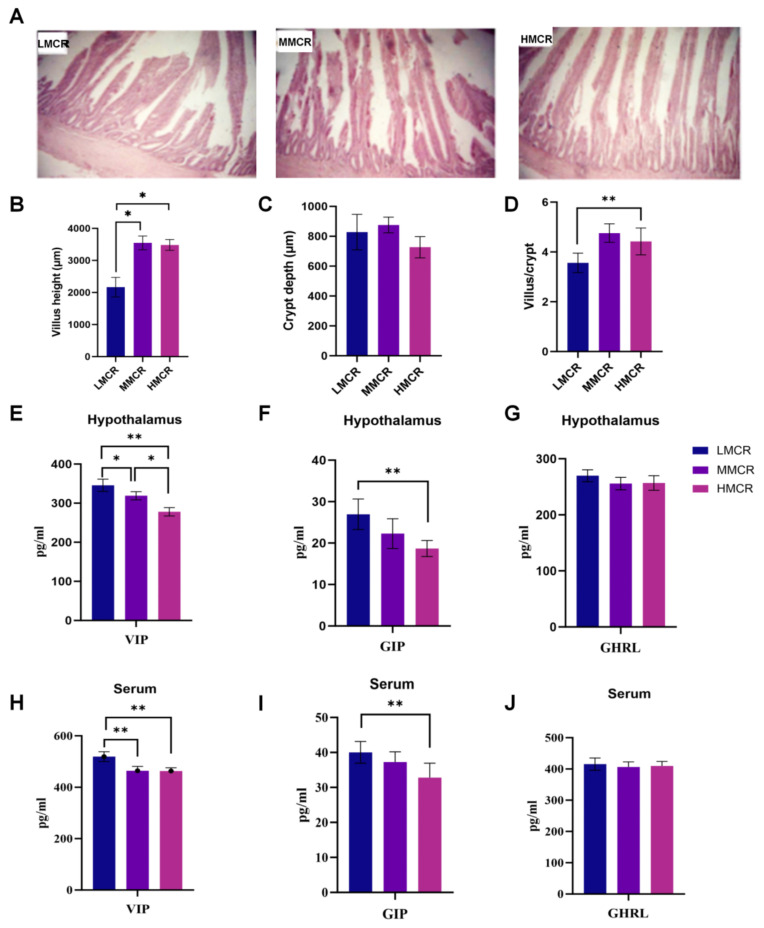
High Met-to-Cys ratio (MCR) levels can promote ileum development while suppressing the secretion of gastrointestinal peptides (n = 3). (A–D) Morphological structure of ileum in 21-day-old broilers (n = 6). (E–G) Effects of MCR on vasoactive intestinal peptide (VIP), gastric inhibitory polypeptide (GIP) and ghrelin (GHRL) in hypothalamic of broilers (n = 6). (H–J) Effects of MCR on VIP, GIP and GHRL in serum of broilers (n = 6). * p<0.05; ** p<0.01. LMCR, low Met-to-Cys ratio; MMCR, middle Met-to-Cys ratio; HMCR, high Met-to-Cys ratio; Met, methionine; Cys, cysteine.

As the ileum is a pivotal organ in the process of digestion and absorption, measurements were taken of morphological changes in the ileum and levels of gastrointestinal hormones were analyzed. Figure 4A shows the effect of different dietary sulfur amino acid ratios on ileum morphology in 21-day-old broiler chickens. Villus height in the ileum was higher in the MMCR and HMCR groups than in the LMCR group (p< 0.05), with no difference between the MMCR and HMCR groups (Figure 4B). The villus-to-crypt ratio was higher in the HMCR group than in the LMCR group (p<0.05). Figures 4H–4J shows the effect of different MCRs on serum gastrointestinal hormone secretion in broiler chickens. At 21 days, the VIP level in the serum of the LMCR group was higher than in the MMCR and HMCR groups (p<0.05). Serum levels of gastric inhibitory polypeptide (GIP) were higher in the LMCR and MMCR groups than in the HMCR group (Figure 4I, p<0.05). In the hypothalamus, the VIP and GIP content was higher in the LMCR than in the MMCR and HMCR (p<0.05), with the MMCR being higher than the HMCR (Figures 4E, 4F, p<0.05).

### The effect of different methionine-to-cysteine ratios on the protein composition of the ileum

**Figure 5:**
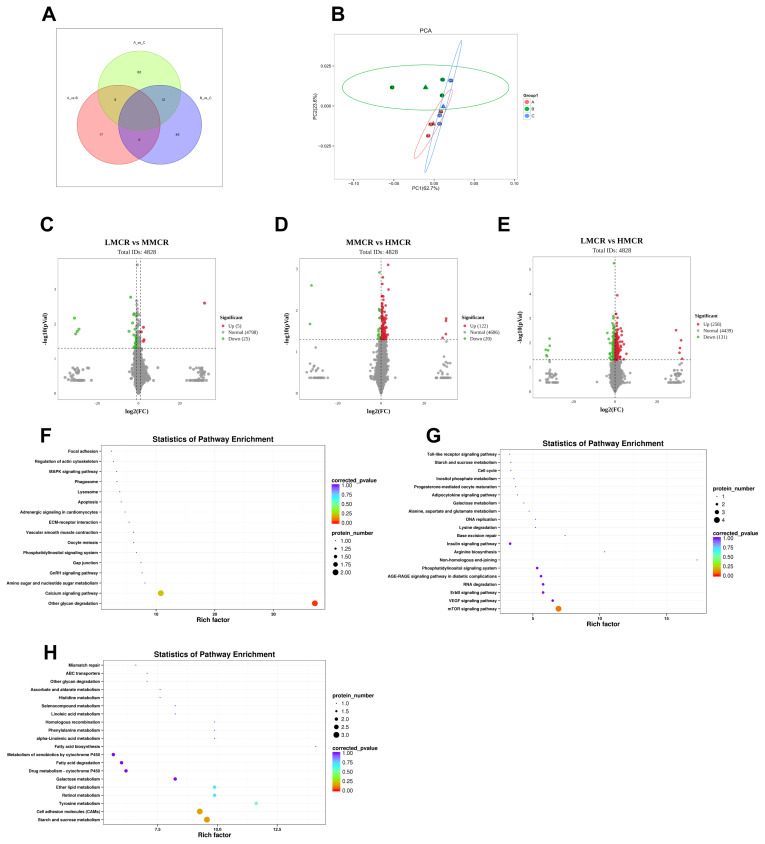
Proteomic analysis of the ileum in broiler chickens under different MCR conditions. (A) Analysis of differential proteins by Venn (n = 3). (B) Analysis of differential proteins by PCA (n = 3). (C–E) Volcano plot of differential protein (n = 3). Red indicates upward adjustments, green indicates downward adjustments. (F–H) KEGG functional classification and clustering results of differential expression proteins (n = 3). * p<0.05; ** p<0.01. MCR, methionine-to- cysteine ratio; KEGG, Kyoto Encyclopedia of Genes and Genomes.

Differential protein expression analysis of the ileum proteins from the LMCR, MMCR and HMCR is shown in Figure 5A and the volcano plot of the differential proteins is shown in Figures 5C–5E. We found that altering the dietary ratio of sulfur-containing amino acids could regulate the differential protein expression in the ileum of broilers. From the volcano plot, we observed that the proportion of differential proteins between the LMCR-MMCR (Figure 5C) and MMCR-HMCR (Figure 5D) in the TPs of the ileum was relatively low. Specifically, compared to LMCR, the differential proteins in HMCR (Figure 5E) amounted to 30, with 5 upregulated and 25 downregulated proteins. Compared to the MMCR group, the HMCR showed 62 differential proteins, with 55 upregulated and 7 downregulated.

### Kyoto Encyclopedia of Genes and Genomes functional annotation of differential proteins in the ileum under different methionine-to-cysteine ratios

The differential proteins in the LMCR-MMCR (Figure 5I) were all downregulated in the KEGG pathways, primarily involving biological processes such as cell processes, environmental information processing, metabolism, and body systems. Among the 15 involved signaling pathways, 7 are related to inositol 1,4,5-trisphosphate receptors (IP3R) (Supplement 5). The differential proteins and their associated KEGG pathways between the MMCR-HMCR (Figure 5J) are analyzed. In the MMCR-HMCR group, except for two downregulated KEGG pathway proteins, the remaining KEGG pathway proteins were upregulated (Supplement 6). Among the 35 involved signaling pathways, 13 are associated with phosphoinositide 3-kinase (PI3K). A total of 43 KEGG pathways are involved in the differential proteins between the LMCR-HMCR (Figure 5K) groups. Among the 43 signaling pathways, nine are related to uridine diphosphate glucuronosyltransferase (UGT), and six are associated with phospholipase A2 (PLA2G). In the LMCR-HMCR group (Supplement 7), nine KEGG pathways associated with UGT were identified.

## DISCUSSION

In recent years, the promotion of low-protein diets has posed challenges in accurately determining the amino acid requirements of animals. Due to the unique relationship between the Met and Cys conversion in poultry, the ratio of these two amino acids in the diet has a significant impact on sulfur amino acid functions. Our study adjusted the MCR while keeping the total sulfur amino acids at the NRC-recommended level of 0.9% for broiler diets. As the proportion of Met increased, both ADG and FCR also increased. The best performance in terms of ADG and FCR was observed when the MCR was 140:100. Interestingly, despite the weight gain, the feed intake did not change with the adjustment of the MCR. We hypothesized that changes in the MCR influenced the metabolic rate of broilers, thereby improving feed efficiency, rather than affecting the central appetite regulation. This suggested that adjusting the MCR in poultry diets could optimize growth performance and feed utilization efficiency, even if feed intake remains constant. These findings contributed to refining dietary formulations in poultry farming to achieve better growth without increasing feed consumption.

We further characterized the biochemical properties of broiler chickens’ serum. As the MCR increased, the levels of TP and ALB in the serum were upregulated. Serum albumin binds and transports both endogenous and exogenous nutrients, and directly reflects the animal’s protein intake [13]. When serum TP and albumin levels increase, the efficiency of protein absorption and utilization in the body is improved [14]. The highest TP and albumin levels were observed when the MCR was 140:100, which also corresponded to the optimal protein utilization efficiency. The TG and TC levels reflect the lipid deposition in the body. An increase in triglyceride levels indicated lipid accumulation. LDL and HDL are key substances involved in lipid metabolism [15,16]. The LDL is responsible for transporting the CHO produced by the liver to various tissues within the body, while HDL facilitates the transfer of CHO from peripheral tissue membranes and plasma to the liver for subsequent metabolic processes. The HDL reduces fat deposition, whereas LDL promotes it [17]. The present study found that the high methionine group (HMCR) exhibited significantly elevated HDL levels, while triglyceride levels decreased with increasing Met concentration. This finding suggests that the fat deposition in the broilers from the high Met-Cys ratio group was lower than in the low ratio group. Concurrently, higher serum GLU levels were observed in the HMCR group compared to the LMCR group (p<0.05). This finding suggests that elevated Met may play a significant role in phospholipid synthesis and hepatic lipid export (VLDL assembly). Collectively, the diminished serum TG and heightened HDL levels mirror the enhanced hepatic lipid metabolic flux in the HMCR group, leading to a reduction in systemic ectopic fat deposition and the promotion of gluconeogenic pathways. This, in turn, contributes to broiler weight gain through enhanced energy utilization, including aerobic GLU oxidation. In conclusion, the present study demonstrates that increasing the Met-Cys ratio has been shown to reduce body fat deposition and increase the efficiency of energy utilization.

The hypothalamus is the central control hub for appetite and energy balance [18]. It integrates peripheral signals such as fat and energy levels and responds accordingly to regulate food intake and energy expenditure. The MCR has been demonstrated to influence serum HDL, LDL, TG, and GLU levels in broilers. Consequently, alterations in the MCR may impact the lipid and protein metabolism levels of broilers, thereby affecting their growth performance. The objective of this study was to investigate whether this phenomenon could be explained by the effects of MCR on hypothalamic hormones. 5-HT, also known as serotonin, is a monoamine neurotransmitter that has been confirmed to upregulate the concentration of brain-derived neurotrophic factor (BDNF), promote the development and protection of neural cells. Immunofluorescence results indicated that as the MCR increased, the BrdU-positive cell number (a marker for neural cell development) also increased, suggesting enhanced development of the hypothalamus. Further research has shown that 5-HT can affect the secretion of other growth hormones. It has been demonstrated by related studies that, in contrast to the observations made in mammals, elevated central 5-HT in poultry does indeed stimulate GH secretion. This, in turn, has been shown to exert a positive regulatory effect on growth hormone [19]. Central serotonergic signaling has an anorexigenic effect by stimulating thermogenesis in brown adipose tissue, thus increasing energy expenditure [20,21]. Conversely, peripheral serotonergic signaling has been demonstrated to promote energy absorption and storage. The increase in MCR was accompanied by elevated levels of both serum and hypothalamic 5-HT, suggesting that the peripheral effects of 5-HT may contribute to the increased body weight of the broiler chickens.

The main difference in the MCR lies in the varying levels of amino acids. To explore the specific changes in the hypothalamus under different MCR, we focused on the impact of the MCR variations on protein metabolism in the hypothalamus. We found that changes in the MCR in the diet did not significantly affect the expression of a large number of proteins. These results indicated the functional differences between Met and Cys. The KEGG analysis revealed that the PI3K pathway, steroid hormones, and neuroreceptor pathways were continuously activated during the MCR changes. Furthermore, the KEGG analysis highlighted that the differential pathways related to body systems and metabolic processes between LMCR and HMCR were mostly associated with PKA, suggesting that PKA is a key protein responding to MCR changes in the hypothalamus. LEP exerts its influence on the central nervous system by suppressing PKA expression, thereby regulating feeding behavior [22], and playing a crucial role in the formation of long-term memory for feeding behavior in chicks [23]. It is hypothesized that increasing the MCR in the diet may alter broiler feeding behavior over the long term by inhibiting PKA expression in the hypothalamus, thereby affecting the regulation of hypothalamic hormone secretion.

A quantitative analysis of the PKA expression in the hypothalamus was conducted, and the results showed significant differences in the PKA protein expression among the three groups. As the MCR increased, the PKA expression was suppressed. The PKA is a cAMP-dependent protein kinase. In the hypothalamus, LEP inhibits PKA activity by affecting the expression of cAMP. Other hormones, such as GIP, also influence the hypothalamic response to hormones by modulating cAMP and its downstream protein expressions. The present study found that as the Met-Cys ratio increased, the expression of gastrointestinal inhibitory polypeptide decreased in broiler chickens. To maintain homeostasis, the content of LEP in the hypothalamus increased in a compensatory manner. This may provide a rationale for why alterations in the MCR did not result in changes to ADFI. Studies suggest that central LEP plays a positive role in GH release induced by GHRH [24]. LEP treatment in rat pituitary primary cells positively affects GHRH-induced GH release. LEP can bind to the GHRH receptor (GHRHR) in the hypothalamus, producing GHRH, thereby promoting GH production [25,26]. Furthermore, LEP-mediated GH metabolism can enhance the metabolic effects of GH. The present study found that an elevated MCR inhibited central LEP function by suppressing PKA and the expression of downstream genes. This results in a compensatory upregulation of hypothalamic LEP levels, which in turn promotes the expression of ghrelin receptors and ghrelin itself. The increase in ghrelin expression and elevation of serum ghrelin levels that results from this process have been demonstrated to promote growth and development.

Elevated levels of growth hormone in the serum prompted an investigation into the developmental status of the ileum, a key site for nutrient absorption. The morphology of the intestine has a decisive impact on the intestinal epithelial surface area [27]. Increased villus height enhances the absorption of nutrients. Intestinal crypts can secrete various digestion-related enzymes. Increasing the villus-to-crypt ratio is beneficial for improving intestinal function [28,29]. As MCR increased, we observed that the villus height in the ileum increased, with no changes in crypt depth, and the FCR was reduced. However, differences were found only between the LMCR and HMCR, which aligns with the changes in lipid metabolism in the blood. Due to changes in central hormones, we characterized gastrointestinal hormones, including GH, which interacts with various other hormones. The NPY is a peptide hormone composed of 36 amino acids and is a crucial appetite-stimulating factor. It inhibits the activity of other neurons in the hypothalamus, such as pro-opiomelanocortin (POMC) neurons, through the release of AgRP and NPY, thereby promoting increased appetite [30–33]. We found that as MCR increased, the concentration of NPY in the hypothalamus decreased, and a similar phenomenon was observed in the serum. However, no significant difference in feed intake was observed in broilers with changes in NPY. The VIP is a peptide that functions both as a hormone and a neurotransmitter [34]. Its primary function is to regulate the relaxation of intestinal smooth muscles. Previous research has demonstrated that an excessively high concentration of VIP can accelerate the emptying of intestinal contents, but it also affects the rapid absorption of nutrients by the intestine. In this experiment, the concentration of VIP in broiler serum was negatively correlated with the MCR. This indicated that when MCR is low, it can accelerate intestinal emptying, thereby reducing the efficiency of nutrient absorption in broilers.

The differences in protein metabolism in the ileum were characterized. Comparative analysis of differentially expressed proteins between the LMCR-MMCR and MMCR-HMCR groups. The MCR regulated the ileum by modulating intercellular signal transduction pathways, thus mediating the response to external signals and influencing the growth and development of broiler chickens. The KEGG pathway analysis revealed that, in the LMCR-HMCR group, differential proteins were associated with 15 signaling pathways, 7 of which were linked to the downregulation of IP3R. In contrast, the MMCR-HMCR group differential proteins were involved in 35 signaling pathways, with 13 pathways related to the upregulation of PI3K. These findings suggest that the regulation is primarily mediated through the two key proteins, IP3R and PI3K. The IP3R is a critical calcium channel regulatory protein within the endoplasmic reticulum that facilitates calcium flow into mitochondria. Studies indicate that under nutrient deficiency conditions, cells downregulate IP3R to activate the MAPK signaling pathway, which triggers autophagy. The autophagic process helps to compensate for cell apoptosis caused by nutrient scarcity [36]. The PI3K is activated by the membrane-bound receptor p85. The SH2 domain of p85 recognizes signals from plasma membrane receptors, and upon recruitment to the membrane, it forms a complex with PI3K that catalyzes a conversion, activating the PI3K signaling pathway [36]. Activation of downstream proteins inhibits ileal apoptosis and promotes ileal cell survival and growth. In the LMCR group, the imbalance of the Met-to-Cys ratio led to the upregulation of IP3R. This upregulation increased mitochondrial calcium uptake and maintained ileal energetic homeostasis, thereby inhibiting autophagy in ileal cells. In circumstances where Met levels are inadequate, there is a selective development of organs involved in nutrient absorption, such as the ileum, in the broiler. However, when Met levels exceed the minimum required for maintenance, the upregulation of IP3R is deregulated and the organism better disperses energy into somatic cells, thereby promoting skeletal muscle development [37,38]. This adaptive mechanism enhances the broiler’s ability to respond to external stressors. Under nutrient-rich conditions, Met fulfills the metabolic demands of various organs and tissues, thereby promoting overall growth performance. It is important to note that broiler chicks undergo significant physiological changes during the neonatal period. In addition to nutritional factors, hormone levels, including brain-gut peptides, are influenced by environmental conditions and sex. The present experiment was conducted in accordance with the nutritional standards for broiler chicks aged 0 to 10 days, as outlined in the AA broiler rearing guidelines. Prior to the commencement of the trial, a pre-feeding period was established, and a gradual transition protocol was implemented for feed changes. This ensured optimal and uniform intestinal and organ development in all chicks prior to the commencement of the trial. To ensure the representation of the findings, a 50:50 male-to-female ratio was employed.

## CONCLUSION

In this study, when the total sulfur amino acid content in the feed was maintained at 0.9%, we found that broiler chickens showed the best growth performance when the MCR was 140:100 (Met:Cys = 0.53%:0.37%). A higher proportion of Met in sulfur-containing amino acids improved the growth performance of broiler chickens. Moreover, a higher proportion of Met enhanced the secretion of brain-gut peptides and increased the expression of growth hormone-related genes by modulating the hypothalamic cAMP pathway. Additionally, changes in MCR improved intestinal development by regulating the expression of IP3R and PI3K in intestinal cells. These findings provide valuable insights into how balancing Met and Cys can enhance the health and growth performance of broiler chickens, offering a foundation for optimizing poultry feed formulations to boost productivity.

## Figures and Tables

**Table 1:** Ingredients, nutrient composition of experimental diets (%, dry matter basis)

Items	LMCR	MMCR	HMCR
Ingredients (%)			
Corn	57.38	57.38	57.38
Soybean meal	37.00	37.00	37.00
Soybean oil	2.32	2.32	2.32
Lysine	0.02	0.02	0.02
Limestone	1.23	1.23	1.23
CaHPO_4_	1.43	1.43	1.43
NaCl	0.19	0.19	0.19
Choline chloride	0.10	0.10	0.10
L-methionine	0.02	0.13	0.21
L-cysteine	0.20	0.09	0.01
Premix^1)^	0.33	0.33	0.33
Total	100.00	100.00	100.00
Nutrient levels (%)^2)^			
Metabolizable energy (MJ/kg)	12.54	12.54	12.54
Organic matter (OM)	96.76	96.76	96.76
Crude protein (CP)	20.79	20.79	20.79
Lysine	1.15	1.15	1.15
Methionine (Met)^3)^	0.34	0.45	0.53
Cysteine (Cys)^3)^	0.56	0.45	0.37
Met:Cys^3)^	60:100	100:100	140:100
Calcium	1.03	1.03	1.03
Available phosphorus	0.46	0.46	0.46

Other essential amino acids, including Thr and Trp, met the recommended requirements of the NRC [5] and were consistent across all treatment groups.

1) Broilers premix could provided per kg of diet: vitamin A, 12,000 IU; vitamin D_3_, 3,500 IU; vitamin E, 40 IU; menadione, 1.3 mg; riboflavin, 7.5 mg; nicotinamide, 41 mg; calcium pantothenate, 10.5 mg; pyridoxine·HCl 4.1 mg; biotin, 0.05 mg; folic acid 1.1 mg; vitamin B_12_, 0.016 mg; Fe, 85 mg; Cu, 7.8 mg; Mn, 105 mg; Zn, 65.5 mg; I, 1.2 mg; Se, 0.4 mg.

2) Metabolizable energy was a calculated value, while the others were measured values.

3) The nutritional values listed were calculated values, while all other nutritional levels are analytical values.

LMCR, low Met-to-Cys ratio; MMCR, middle Met-to-Cys ratio; HMCR, high Met-to-Cys ratio.

**Table 2:** The real-time PCR primers

Gene	GenBank ID	Primer sequences (5′ to 3′)
*GAPDH*	NM_204305.1	F: ATGCTTCTAGGCGGACTGT R: CCATCCAACCGACTGCT
*GHRH*	NM_001040464.1	F: TTTTCACCGACAACTACCG R: GCTCCCAAGAAGTCCCTC
*GHRHR*	NM_001201396.1	F: TAGGTCGGAGATGGC R: CAGATTCGGAGGTTGTC’
*GH*	NM_204359.2	F: CCTGCTGTCCTGCTTCAA R: GGGTTTATTCCTCGTGTTTT

PCR, polymerase chain reaction.

## Data Availability

Upon reasonable request, the datasets of this study can be available from the corresponding author.

## SUPPLEMENTARY MATERIAL

**Supplement 1.** Arbor Acres broiler starter diet nutritional analysis.

**Supplement 2.** Analysis of KEGG pathway of differential protein in Hypothalamus of groups low Met:Cys ratio (LMCR) and middle Met:Cys ratio (MMCR).

**Supplement 3.** Analysis of KEGG pathway of differential protein in Hypothalamus of groups middle Met:Cys ratio (MMCR) and high Met:Cys ratio (HMCR).

**Supplement 4.** Analysis of KEGG pathway of differential protein in Hypothalamus of groups middle Met:Cys ratio (MMCR) and high Met:Cys ratio (HMCR).

**Supplement 5.** Analysis of KEGG pathway of differential protein in ileum of groups low Met:Cys ratio (LMCR) and middle Met:Cys ratio (MMCR).

**Supplement 6.** Analysis of KEGG pathway of differential protein in ileum of groups middle Met:Cys ratio (MMCR) and high Met:Cys ratio (HMCR).

**Supplement 7.** Analysis of KEGG pathway of differential protein in ileum of groups low Met:Cys ratio (LMCR) and high Met:Cys ratio (HMCR).
