# Supplementary material for: Effects of different dietary methionine and cysteine ratios on growth performance and intestinal development of broilers from brain-gut peptide secretion perspective
Source: Anim Biosci. 2026 Feb 6;39(6):250787. doi: 10.5713/ab.250787 (PMC13243930; doi:10.5713/ab.250787)
Supplement: Supplementary file 1 [file ab-250787-Supplementary-1.pdf]

Supplement 1. Arbor Acres Broiler Starter Diet Nutritional Analysis.

Items	Control group
**Ingredients (%)**	
Corn	54.20
Soybean meal	38.50
Soybean oil	2.80
Dicalcium phosphate	1.90
Limestone	1.30
Sodium chloride	0.30
DL-Methionine	0.28
L-Lysine HCl	0.12
Choline chloride (50%)	0.10
Premix[1]	0.50
Total	100.00
**Calculated Nutrient Levels[2]**	
Metabolizable Energy (ME)	12.55 MJ/kg
Crude Protein (CP)	23.00 %
Calcium (Ca)	1.00 %
Available Phosphorus (AP)	0.48 %
Lysine (Lys)	1.28 %

[1] The premix provided the following per kg of diet: VA 12,000 IU, VD3 3,000 IU, VE 30 mg, VK3 2 mg, VB1 2 mg, VB2 6 mg, VB6 4 mg, VB12 0.02 mg, Biotin 0.15 mg, Folic acid 1 mg, Niacin 40 mg, Pantothenic acid 12 mg, Fe 80 mg, Cu 8 mg, Zn 75 mg, Mn 100 mg, I 0.35 mg, Se 0.15 mg.

[2] Nutrient levels were calculated values based on the ingredient composition.
