# Supplementary material for: Effects of different dietary methionine and cysteine ratios on growth performance and intestinal development of broilers from brain-gut peptide secretion perspective
Source: Anim Biosci. 2026 Feb 6;39(6):250787. doi: 10.5713/ab.250787 (PMC13243930; doi:10.5713/ab.250787)
Supplement: Supplementary file 2 [file ab-250787-Supplementary-2.pdf]

Supplement 2. Analysis of KEGG pathway of differential protein in Hypothalamus of groups low Met:Cys ratio (LMCR) and middle Met:Cys ratio (MMCR).

Pathway ID	Pathway name	Upgrade expression proteins	Degrade expression proteins
ko05164	Influenza A		TRIM 25
ko03013	RNA Transport		RANBP2; EIF2B
ko03015	mRNA surveilance pathway		PP2A
ko03320	PPAR signaling pathway	CYP27	
ko04020	Calcium signaling pathway	ROC; PDEL	
ko04080	Neuroactive ligand-receptor interaction	GRIN2A	
ko04114	Oocyte meiosis		B56
ko04120	Ubiquitin mediated proteolysis	ARF-BP1	
ko04130	SNARE interactions in vesicular transport	STX8	
ko04141	Protein processing in endoplasmic reticulum		CALPAIN
ko04142	Lysosome	Cln7	MPR
ko04144	Endocytosis	ARFGEF	
ko04145	Phagosome		M6PR
ko04146	peroxisome		PEX11
ko04210	Apoptosis		CALPAIN
ko04261	Adrenergic signaling in cardiomyocytes		PP2A
ko04270	Vascular smooth muscle contraction	PKG	
ko04310	Wnt signaling pathway		TBL1
ko04510	Focal adhesion		ECM
ko04512	Ecm-receptor interaction		LAMININ
ko04520	Adherene junction		YES
ko04540	Gap junction	PKG	
ko04622	RIG -I-like receptor signaling pathway		TRIM 25
ko04810	Regulation of actin cytoskeletor	LIMK	
ko00100	Steroid Biosynthesis		HSD17B7
ko00120	Primarybile acid biosyhthesis	CYP27A1	
ko00140	Steroid Hormone Biosynthesis		HSD17B7
ko00230	Purine Metabolism	PDE1	
ko00450	Selenocompound Metabolism		SEPHS
ko00564	Glycerophospholipid Metabolism	CDS1	
ko00860	Porphyrin and Chlorophyll Metabolism		FECH
ko00920	Sulfur Metabolism		ETHE1
ko04070	Phosphatidylinositol Signaling System	CDS1	
