# Supplementary material for: Effects of different dietary methionine and cysteine ratios on growth performance and intestinal development of broilers from brain-gut peptide secretion perspective
Source: Anim Biosci. 2026 Feb 6;39(6):250787. doi: 10.5713/ab.250787 (PMC13243930; doi:10.5713/ab.250787)
Supplement: Supplementary file 3 [file ab-250787-Supplementary-3.pdf]

Supplement 3. Analysis of KEGG pathway of differential protein in Hypothalamus of groups middle Met:Cys ratio (MMCR) and high Met:Cys ratio (HMCR).

Pathway ID	Pathway name	Upgrade expression proteins	Degrade expression proteins
ko00590	Arachidonic acid metabolism	CYP2C	
ko04070	Phosphatidylinositol signaling system	PI4K	
ko00591	Linoleic acid metabolism	Cyp2C	
ko04080	Neuroactive ligand-receptor interaction	EDGL	
ko04115	p53 signaling pathway		PIRH-2
ko04120	Ubiquitin mediated proteolysis		PIRH-2
ko04145	Phagosome		α2β1
ko04510	Focal adhesion	COL4A	ITGA2
ko04512	ECM-receptor interaction	COL4A	ITGA2
ko04540	Gap junction	EDG2	
ko04622	RIG-I-like receptior signaling pathway	TRIM25	
ko04810	Regulation of actin cytoskeleton		ITG
ko04933	AGE-RAGE signaling pathway in diabetic complications	COL	
ko05164	Influenza A	TRIM25	
ko00140	Steroid Hormone Biosynthesis	COL4A	
ko00220	Arginine Biosynthesis		TRIM25
ko00240	Pyrimidine Metabolism	CYP2C	
ko00330	Arginine and Proline Metabolism		rocF
ko00520	Amino Sugar and Nucleotide Sugar Metabolism	GNPDA	
ko00670	One carbon Pool by Folate	DHFR	
ko00790	Folate Biosynthesis	DHFR	
ko00830	Retinol Metabolism in Animals	CYP2C	
ko00562	Inositol Phosphate Metabolism	PI4K2	
