# Supplementary material for: Effects of different dietary methionine and cysteine ratios on growth performance and intestinal development of broilers from brain-gut peptide secretion perspective
Source: Anim Biosci. 2026 Feb 6;39(6):250787. doi: 10.5713/ab.250787 (PMC13243930; doi:10.5713/ab.250787)
Supplement: Supplementary file 4 [file ab-250787-Supplementary-4.pdf]

Supplement 4. Analysis of KEGG pathway of differential protein in Hypothalamus of groups middle Met:Cys ratio (MMCR) and high Met:Cys ratio (HMCR).

Pathway ID	Pathway name	Upgrade expression proteins	Degrade expression proteins
ko00100	Steroid biosynthesis	DHCR7	HSD17B7
ko00590	Arachidonic acid metabolism	CYP2C	
ko00591	Linoleic acid metabolism	Cyp2C	
ko00592	α-Linolenic acid metabolism	CYP2C	
ko03010	Ribosome	MRPL22; MRPL4	RPL15
ko03013	RNA transport	eIF2B; Nup133; Nup53	PYM
ko03015	mRNA surveillance pathway	PYM	
ko03018	RNA degradation	PfkA	
ko03040	Spliceosome	PPIE; SF3B2	TRA2
ko03060	Protein export	SEC63	
ko03320	PPAR signaling pathway	ApoA2	ACOX1
ko03440	Homologous recombination		Top3
ko03460	Fanconi anemia pathway		Top3
ko04152	MAPK signaling pathway	PKA; Tau	CACNA1B
ko04012	ErbB signaling pathway	GAB1	
ko04020	Calcium signaling pathway	PKA; CD38	CACNA1B
ko04022	cGMP-PKG signaling pathway		MLC
ko04024	cAMP signaling pathway		MLC
ko04068	FoxO signaling pethway		AMPK
ko04110	Cell cycle	APC7	CHEK2; SMC1
ko04114	Oocyte meiosis	PKA; APC7	ADCY5; SMC1
ko04115	P53 signaling pathway		CHK2
ko04120	Ubiquitin mediated proteolysis	Apc7	
ko04141	Protein processing in endoplasmic reticulum	SEC63	SEC24; CAPN1
ko04142	Lysosome	NPC1; AP1S1_2	PSAP
ko04144	Endocytosis	ACAP; CYTH; ASAP	SH3GL
ko04145	Phagosome	TUBA	ITGA2
ko04146	Peroxisome	PEX11A	PEX11B/ACOX1
ko04151	PI3K-AKT signaling pathway		CYS
ko04010	AMPK signaling pathway		GS
ko04210	Apoptosis	TUBA	CAPN1

ko04261	Adrenergic signaling in cardiomyocytes	PKA	ADCY5
ko04270	Vascular smooth muscle contraction	PKA; PRKG1	ADCY5; MYL9
ko04310	Wnt signaling pathway	PKA	
ko04340	Hedgehog signaling pathway	PKA	CSNK1G
ko04510	Focal adhesion		MYL9; ITGA2
ko04512	Ecm-receptor interaction		ITGA2
ko04530	Tight junction	EPB41	MYL9
ko04540	Gap junction	TUBA; PKA; PRKG1	ADCY5
ko04670	Leukocyte transendothelial migration		MYL9
ko04810	Regulation of actin cytoskeleton	LIMK1	MYL9; ITGA2
ko04910	Insulin signaling pathway	PKA	PRKAG; GYS
ko04912	GnRH signaling pathway	PKA	ADCY5
ko04914	Progesterone-mediated oocyte maturation	PKA; APC7	ADCY5
ko04916	Melanogenesis	PKA	ADCY5
ko04920	Adipocytokine signaling pathway		AMPK
ko04921	Oxytocin signaling pathway		MYL9
ko04922	Glucagon signaling pathway		GYS; PPP4C
ko04931	Insulin resistance		GYS
ko05205	Proteoglycans in cancer	ANK1	
