# Supplementary material for: Effects of different dietary methionine and cysteine ratios on growth performance and intestinal development of broilers from brain-gut peptide secretion perspective
Source: Anim Biosci. 2026 Feb 6;39(6):250787. doi: 10.5713/ab.250787 (PMC13243930; doi:10.5713/ab.250787)
Supplement: Supplementary file 5 [file ab-250787-Supplementary-5.pdf]

Supplement 5. Analysis of KEGG pathway of differential protein in ileum of groups low Met:Cys ratio (LMCR) and middle Met:Cys ratio (MMCR).

Pathway ID	Pathway name	Upgrade expression proteins	Degrade expression proteins
ko00520	Amino Sugar And Nucleotide Sugar Metabolism		GicNAc
ko04070	Phosphatid Ylinositol Signaling		IP3R
ko04114	Oocyte Meiosis		IP3R
ko04210	Apoptosis		IP3R
ko04270	Vascular Smooth Muscle Contraction		IP3R
ko04540	Gap Junction		IP3R
ko04912	Gnrh Signaling Pathway		IP3R
ko04020	Calciun Signaling		IP3R、PMCA
ko04810	Regulation of Actin Cytoskeleton		ITG
ko04510	Focal Adhesion		ITGA
ko04010	Mapk Signaling		NFαB
ko04261	Adrenergic Signaling In Cardiomyocytes		PMCA
ko04142	Lysosome		TPP1
ko04512	Ecm-Receptor Interaction		α2
ko04145	Phagosome		α2β1
