# Supplementary material for: Effects of different dietary methionine and cysteine ratios on growth performance and intestinal development of broilers from brain-gut peptide secretion perspective
Source: Anim Biosci. 2026 Feb 6;39(6):250787. doi: 10.5713/ab.250787 (PMC13243930; doi:10.5713/ab.250787)
Supplement: Supplementary file 6 [file ab-250787-Supplementary-6.pdf]

Supplement 6. Analysis of KEGG pathway of differential protein in ileum of groups middle Met:Cys ratio (MMCR) and high Met:Cys ratio (HMCR).

Pathway ID	Pathway name	Upgrade expression proteins	Degrade expression proteins
ko03010	Ribosome	--	L27Ae
ko04120	Ubiquitin mediated proteolysis	--	TRIP12
ko00310	Lysine degradation	MLL2	
ko00520	Amino sugar and nucleotide sugar metabolism	NAGK	
ko00562	Inositol phosphate metabolism	PLCG2	
ko00500	Starch and sucrose metabolism	GAA	
ko00052	Galactose metabolism	GAA	
ko00220	Arginine biosynthesis	ARGH	
ko00250	Alanine, aspartate and glutamate metabolism	ARGH	
ko03030	DNA replication	Fen1	
ko04142	Lysosome	GAA	
ko04512	ECM-receptor interaction	Laminin	
ko04010	MAPK signaling pathway	NFkB	
ko03015	mRNA surveillance pathway	PAP	
ko04620	Toll-like receptor signaling pathway	PI3K	
ko04810	Regulation of actin cytoskeleton	PI3K	
ko04068	FoxO signaling pathway	PI3K	
ko04210	Apoptosis	PI3K	
ko04914	Progesterone-mediated oocyte maturation	PI3K	
ko05164	Influenza A	PI3K	
ko04510	Focal adhesion	PI3K、ECM	
ko04012	ErbB signaling pathway	PI3K、PLC	
ko04933	AGE-RAGE signaling pathway in diabetic complications	PI3K、PLC	
ko04370	VEGF signaling pathway	PI3K、PLCγ	
ko04910	Insulin signaling pathway	PI3K、Rheb	
ko04150	mTOR signaling pathway	PI3K、V-ATPase、GAToR2、Rheb	
ko04070	Phosphatidylinositol signaling system	PIK3、PLC	
ko04270	Vascular smooth muscle contraction	PKC	
ko04920	Adipocytokine signaling pathway	PKCθ	
ko04020	Calcium signaling pathway	PLCγ	
ko03018	RNA degradation	Prp40	
ko04110	Cell cycle	Rad21	

ko03450	Non-homologous end-joining	Rad27
ko04145	Phagosome	VATPase
