# Supplementary material for: Effects of different dietary methionine and cysteine ratios on growth performance and intestinal development of broilers from brain-gut peptide secretion perspective
Source: Anim Biosci. 2026 Feb 6;39(6):250787. doi: 10.5713/ab.250787 (PMC13243930; doi:10.5713/ab.250787)
Supplement: Supplementary file 7 [file ab-250787-Supplementary-7.pdf]

Supplement 7. Analysis of KEGG pathway of differential protein in ileum of groups low Met:Cys ratio (LMCR) and high Met:Cys ratio (HMCR).

Pathway ID	Pathway name	Upgrade expression proteins	Degrade expression proteins
ko00340	Histidine metabolism	DDC	
ko00010	Glycolysis / Gluconeogenesis	ADH1/7	
ko04920	Adipocytokine signaling pathway		ACSL
ko00230	Purine metabolism	ENTPD5/6	
ko00140	Steroid hormone biosynthesis	UGT	
ko00590	Arachidonic acid metabolism	PLA2G	
ko00450	Selenocompound metabolism	SCLY	
ko00592	alpha-Linolenic acid metabolism	PLA2G	
ko00240	Pyrimidine metabolism	ENTPD5_6	
ko03030	DNA replication	SSB	
ko03440	Homologous recombination	SSB	
ko00564	Glycerophospholipid metabolism	PLA2G	
ko00511	Other glycan degradation		HEX
ko00860	Porphyrin and chlorophyll metabolism	UGT	
ko00983	Drug metabolism - other enzymes	UGT	
ko00360	Phenylalanine metabolism	DDC	
ko00061	Fatty acid biosynthesis		ACSL
ko00053	Ascorbate and aldarate metabolism	UGT	
ko02010	ABC transporters	ABCC2	
ko00591	Linoleic acid metabolism	PLA2G	
ko04270	Vascular smooth muscle contraction	PLA2G	
ko03430	Mismatch repair	SSB	
ko04146	Peroxisome		ACSL
ko03040	Spliceosome	BCAS2	
ko00600	Sphingolipid metabolism	CGT	
ko03320	PPAR signaling pathway		ACSL
ko00040	Pentose and glucuronate interconversions	UGT	
ko00380	Tryptophan metabolism	DDC	
ko01212	Fatty acid metabolism		ACSL
ko03013	RNA transport		RANBP2
ko00071	Fatty acid degradation	ADH1/7	ACSL
ko04144	Endocytosis	MHC1	DAB2
ko04142	Lysosome	M6PR, GAA	
ko00052	Galactose metabolism	MGAM, GAA	
ko05168	Herpes simplex infection	MHC1, C5	
ko00980	Metabolism of xenobiotics by cytochrome P450	ADH1_7, UGT	

ko04145	Phagosome	M6PR, MHC1	
ko00565	Ether lipid metabolism	CGT, PLA2G	
ko00982	Drug metabolism - cytochrome P450	ADH1/7, UGT	
ko00830	Retinol metabolism	ADH1/7, UGT	
ko00350	Tyrosine metabolism	DDC, ADH1/7	
ko04514	Cell adhesion molecules (CAMs)	MHC1	PECAM1, CD99
ko00500	Starch and sucrose metabolism	UGT, MGAM, GAA	
